# Therapeutic Effects of *Phyllanthus urinaria L* on Cisplatin‐Induced Acute Kidney Injury: Anti‐Inflammatory, Anti‐Apoptotic, and Anti‐Oxidative Action

**DOI:** 10.1002/pdi3.70000

**Published:** 2025-05-15

**Authors:** Qitong Guo, Ping Li, Meiling Chen, Yihang Yu, Lianju Shen, Chunlan Long, Xing Liu, Guanghui Wei, Deying Zhang

**Affiliations:** ^1^ Department of Urology Children's Hospital of Chongqing Medical University Chongqing China; ^2^ Ministry of Education Key Laboratory of Child Development and Disorders Chongqing Key Laboratory of Structural Birth Defect and Reconstruction National Clinical Research Center for Child Health and Disorders China International Science and Technology Cooperation Base of Child Development and Critical Disorders Chongqing China

**Keywords:** apoptosis, cisplatin‐induced acute kidney injury(AKI), inflammation, oxidative stress, *Phyllanthus urinaria L*

## Abstract

Acute kidney injury (AKI) is a prevalent and serious clinical challenge with limited specific treatments. *Phyllanthus urinaria L* (*PU*), a well‐regarded herbal medicine, possesses noted anti‐inflammatory, antiviral, and antitumor properties. Yet, research into its renal protective effects, particularly for cisplatin‐induced AKI, is markedly limited. Our study explores the therapeutic potential of *PU* against AKI triggered by cisplatin administration. Utilizing an AKI model induced by a substantial dose of cisplatin, we assessed renal function through histological examination (hematoxylin and eosin staining) and biochemical markers, including blood urea nitrogen (BUN) and serum creatinine (Scr). Our results demonstrated that *PU* may mitigate cisplatin's structural damage to renal tissue. Post‐treatment with *PU*, a significant reduction in pro‐inflammatory cytokines interleukin‐1β (IL‐1β), IL‐6, and tumor necrosis factor‐α (TNF‐α) was observed through quantitative polymerase chain reaction (PCR) analysis. Additionally, the expressions of apoptotic and anti‐apoptotic proteins B cell leukemia/lymphoma two (Bcl‐2), Bcl2‐Associated X protein (Bax), and cleaved‐caspase3 evaluated via Western blot and immunofluorescence indicated that *PU* could inhibit apoptosis in response to cisplatin injury. Oxidative stress markers, such as malondialdehyde (MDA) and superoxide dismutase (SOD), showed decreased MDA levels and elevated SOD activity following *PU* treatment, suggesting a reduction in oxidative damage. Furthermore, cisplatin‐induced downregulation of *NRF2* and *NQO1* was countered by *PU*, highlighting its restorative effect on antioxidant pathways as confirmed by Western blot and immunofluorescence. Collectively, our findings endorse *PU*'s protective capability against cisplatin‐induced AKI, endorsing its therapeutic potential to counteract cisplatin's nephrotoxicity.

## Graphical abstract

*Phyllanthus urinaria L* can alleviate cisplatin‐induced acute kidney injury by inhibiting inflammation, apoptosis and oxidative stress.

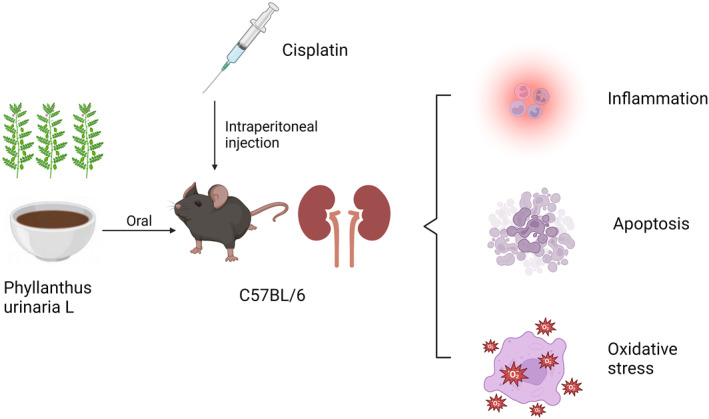

## 1 Introduction

Acute kidney injury (AKI) is a dynamic disorder characterized by a rapid decline in renal function, triggered by various etiologies including hemorrhagic shock, direct renal trauma, massive blood transfusion, and nephrotoxic medications [1]. AKI prevalence in hospitalized patients ranges widely, from 10% to 15%, escalating to over 50% in intensive care units [2]. Annually, AKI contributes to approximately 2 million deaths globally because of its high incidence and dismal prognosis with a post‐90‐day mortality rate reaching up to 28% [3]. In China, AKI occurs with an alarming prevalence of 11.6%, posing significant threats to public health and socioeconomic stability [4].

Pharmaceuticals rank among the common culprits of AKI in a clinical setting, accounting for 14%–26% of adult cases [5, 6], and similar trends are observed in pediatric AKI. Notably, cisplatin, one of broadly applied chemotherapeutics for solid tumors, is synonymous with nephrotoxicity—its principal dose‐dependent side effect. Cisplatin's accumulation in renal tubular epithelial cells triggers apoptosis, inflammatory responses, and oxidative stress [7] leading to AKI in nearly 30% of recipients [8]. Murine models of AKI, induced via a single large dose of cisplatin (20–30 mg/kg), mirror the human condition with pronounced renal tubular damage, often magnified by concomitant inflammation and vascular injury [9]. Current AKI treatments predominantly involve renal replacement therapy due to a lack of effective pharmaceutical interventions [10]; thus, exploring accessible and efficacious preventative measures and treatments is imperative.

In the quest for renal protective strategies, natural herbs, exemplified by ginseng, curcumin, and pomegranate, have shown considerable effectiveness as natural antioxidants and anti‐inflammatory agents against AKI [11]. Among these, *Phyllanthus urinaria L* (*PU*), part of the *Euphorbiaceae* family, is celebrated in traditional Chinese medicine, boasting a legacy of treating diverse conditions such as cancer, liver disease, cardiovascular disorders, and diabetes [12, 13]. *PU* and its compound preparations can reduce immune and chemical induced liver injury and play a protective role in liver, and the hepatoprotective mechanism of *PU* may be related to the scavenging of oxygen free radicals and the inhibition of lipid oxidation reaction [14]. *PU* can induce the apoptosis of cancer cells, inhibit the proliferation of cancer cells, and have growth inhibitory activity on different types of cancer cells without affecting the growth of normal cells [13]. *PU* alcohol extract has antioxidant and cardioprotective effects on doxorubicin toxicity in H9C2 myoblast cells [15]. *PU* can play a hypoglycemic role by inhibiting α‐glucosidase [16]. Despite extensive data demonstrating the significant therapeutic effects of *PU* in various diseases, its role in AKI has not been specifically explored. This study, therefore, endeavors to elucidate the impact of *PU* on cisplatin‐induced AKI and envision novel prevention and treatment methodologies.

## 2 Method and material

### 2.1 Preparation of Aqueous Extract From *PU*

*PU* was purchased from Yunnan Hongxiang Traditional Chinese Medicine Technology Co. Ltd. The whole plant powder of aqueousness was soaked in distilled water for 1 h and then boiled at 100% heat until boiling. After boiling, the heat was reduced to medium‐low and continued to heat for 1 h. After cooling for 30 min, the extract was filtered. The residue was repeated twice using the same method, and then the filtrates were combined and filtered repeatedly using 0.45 and 0.22 μm filters. Finally, the concentration of the extract was adjusted to 320 mg/mL with water and stored sealedly at 4°C.

### 2.2 Experimental Animals

The Laboratory Animal Center of Chongqing Medical University (license number: SYXK [Chongqing] 2022–0016) furnished 24 male C57BL/6 mice, 8 weeks old and weighing between 18 and 25 g. The mice were housed in the Experimental Animal Center of Chongqing Children's Hospital (license number: SYXK [Chongqing] 2022–0002), with free access to food and water, and a temperature range of 20°C–25°C and a relative humidity of 40%–60%. Twenty‐four mice were split into three groups at random: control group, cisplatin group, and cisplatin+*PU* group (250 mg/kg). Mice in the cisplatin group received a single intraperitoneal injection of cisplatin (Sigma, Peoria, Illinois, USA) at a dose of 20 mg/kg. When cisplatin was injected intraperitoneally, mice in the Cisplatin+*PU* group were gavaged orally with 250 mg/kg of *PU* for 3 days in a row (days 0 through day 2). The mice in the other two groups were gavaged orally with the equivalent volume of phosphate buffer saline (PBS) for three times in total. On day 4, the mice were subjected to euthanasia, following that their blood samples were obtained for the purpose of biochemical examination. Samples of kidney tissue were taken in order to perform relevant index detection.

### 2.3 Determination of Blood Urea Nitrogen (BUN) and Serum Creatinine (Scr)

After anaesthetization of the mice with isoflurane, blood samples were obtained and centrifuged at 4°C for 10 min to obtain serum. Immediately, the level of serum BUN and Scr were measured using commercial kits (Jiancheng, Nanjing, Jiangsu, China), according to the manufacturer's instructions.

### 2.4 Measurement of Antioxidant Enzyme Activity

Kidney tissue was weighed, added to cold lysis buffer (Beyotime, Shanghai, China), and mechanically homogenized at 1000 rpm for 15 min at 4°C, and the activities of malondialdehyde (MDA) and superoxide dismutase (SOD, Beyotime, Shanghai, China) were measured according to the commercial kits manufacturer's instructions.

### 2.5 Histological Examination

The collected kidney tissue was fixed with 4% paraformaldehyde, and then embedded, sectioned, and deparaffinized. The 4 μm paraffin sections of the kidney tissue were stained with hematoxylin and eosin (HE) and observed under a light microscope.

### 2.6 Quantitative Polymerase Chain Reaction (qPCR)

Kidney tissues were subjected to total RNA extraction utilizing TRIzol reagent (Invitrogen, Carlsbad, California, USA) following the manufacturer's instructions. Subsequently, 1 μg of total RNA was reverse‐transcribed into cDNA using RT master mix for qPCR (gDNA digester plus, MCE, New Jersey, USA), serving as the template for PCR amplification. Gene mRNA levels were detected using SYBR Green master mix (MCE, Kenilworth, New Jersey, USA). The primer sequences employed are presented in Supporting Information S1: Table 1. GAPDH was employed as the internal reference, and the relative quantification method (2^‐∆∆Ct^) was employed for gene quantification.

### 2.7 Immunofluorescence

Paraffin sections of the kidney tissue were obtained following the aforementioned procedure. After deparaffinization, the sections were subjected to antigen retrieval using citrate buffer, and endogenous peroxidase was blocked with 3% H_2_O. The sections were then washed three times with PBS, blocked using 5% bovine serum albumin (Solarbio, Beijing, China) and 0.1% Triton X‐100 (Solarbio, Beijing, China) for 1 h, and incubated overnight at 4°C with primary antibodies against Neutrophil Gelatinase‐Associated Lipocalin (NGAL) (1:200, Proteintech, Wuhan, Hubei, China), cleaved‐caspase3 (1:200, Proteintech, Wuhan, Hubei, China), Bax (1:200, Proteintech, Wuhan, Hubei, China), Bcl‐2 (1:200, Proteintech, Wuhan, Hubei, China), *NRF2* (1:200, ABclonal, Wuhan, Hubei, China), and *NQO1* (1:200, ABclonal, Wuhan, China). After washing the sections three times with PBS, the appropriate fluorescent secondary antibodies (1:200, Proteintech, Wuhan, Wuhan, China) were applied for 1 h. Finally, DAPI staining (1:200, Beyotime, Shanghai, China) was conducted to label the cell nuclei. The acquired images were captured using a confocal microscope (Nikon, Tokyo, Japan) and subsequently analyzed and processed using Nis Viewer software.

### 2.8 Western Blot

Western blot was performed to detect the expression of proteins involved in renal injury, apoptosis, and the *NRF2*/*KEAP1* signaling pathway. Kidney tissue stored at −80°C was lysed using RIPA buffer (MCE, Kenilworth, New Jersey, USA) which included 1% phenylmethylsulfonyl fluoride (PMSF, MCE, Kenilworth, New Jersey, USA). The lysate was then sonicated for 10 min at 4°C and centrifuged at 1000 rpm for 20 min at 4°C. Protein concentration was measured using the BCA assay kit (Beyotime, Shanghai, China). Each protein sample was mixed with 5 ×  buffer at a ratio of 4:1 and boiled for 10 min to obtain the desired protein samples. The protein samples were separated using 7.5%–10% SDS‐PAGE gel electrophoresis (EpiZyme, Shanghai, China), followed by transfer onto polyvinylidene fluoride (PVDF) membranes (Millipore, Boston, Massachusetts, USA). After blocking the membrane with blot‐blocking buffer (NCM, Suzhou, Jiangsu, China) for 10 min, the membrane was incubated with primary antibodies against anti‐NGAL (1:1000, Proteintech, Wuhan, Hubei, China), anti‐cleaved‐caspase3 (1:1000, Proteintech, Wuhan, Hubei, China), anti‐Bax (1:1000, Proteintech, Wuhan, Hubei, China), anti‐Bcl‐2 (1:1000, Proteintech, Wuhan, Hubei, China), anti‐NRF2 (1:1000, Proteintech, Wuhan, Hubei, China), and anti‐NQO1 (1:1000, Proteintech, Wuhan, Hubei, China) at 4°C for 12 h. The membrane was then washed three times with TBST and incubated with goat anti‐mouse or goat anti‐rabbit G secondary antibodies (Zhongshan, Beijing, China) for 1 h. Protein bands were visualized using an enhanced chemiluminescence substrate (Thermo Scientific, Waltham, Massachusetts, USA), and the images were captured using the ECL kit (Bio‐Rad, CA, USA). The Western blot experiment was repeated at least three times.

### 2.9 Data Analysis

Data were analyzed using GraphPad Prism 8.0 (GraphPad Software, San Diego, California, USA). One‐way analysis of variance (ANOVA) was used to compare the differences between experimental and control groups, and post‐hoc Tukey test was used for multiple comparisons. Results were presented as mean ± standard error of the mean (SEM), and p < 0.05 was considered statistically significant.

## 3 Results

### 3.1 PU Ameliorates Renal Dysfunction and Structural Damage in Cisplatin Induced AKI Mice

**FIGURE 1:**
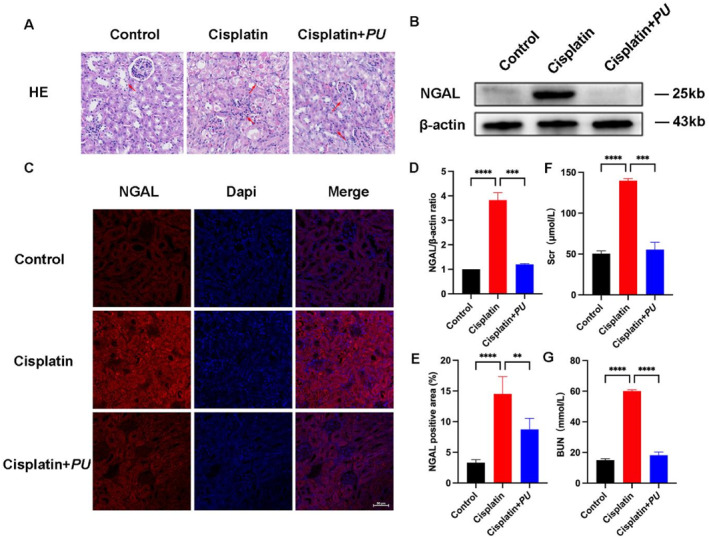
Examination of kidney morphology, renal function, and renal injury marker in the control group, cisplatin group, and cisplatin+*PU* group. (A) Histopathological staining of kidney sections from each group with HE (scale bar: 25 μm). (B) Western blot of NGAL in each group. (C) Immunofluorescence of NGAL in the kidney sections in each group (scale bar: 50 μm). (D) Relative expression of NGAL detected by western blot. (E) Numerical evaluation of immunofluorescence staining for NGAL in each group. (F and G) Levels of biochemical parameters (Scr and BUN) in each group in comparison to the cisplatin group. **p < 0.005, ***p < 0.0005, and ****p < 0.0001. HE, hematoxylin and eosin; NGAL, Neutrophil Gelatinase‐Associated Lipocalin; Scr, Serum Creatinine; BUN, blood urea nitrogen.

The survival rate of all mice within each experimental group was observed to be 100%. This suggests that the administration of high‐dose cisplatin in isolation did not result in any fatalities, and that the intervention including *PU* did not induce mortality in the mice. Renal function was measured through the measurement of Scr and BUN levels in all experimental groups in order to assess any potential protective effects of *PU* against cisplatin‐induced AKI (Figure 1 F, G). Scr and Bun levels in the cisplatin group were significantly higher compared to the control group and the *PU*+cisplatin group, suggesting a severe decline in renal function in the cisplatin group. This finding further suggested that the AKI model was successfully established. When the kidney's structure was examined using HE staining, it was discovered that the renal tubular structures in the cisplatin group were noticeably more disorganised than those in the control and *PU*+cisplatin groups with an infiltration of inflammatory cells (Figure 1 A). Cisplatin significantly increased NGAL expression compared to control and *PU*+cisplatin groups (Figure1 B–E). There is no doubt that cisplatin impairs the function and structure of the kidney, although *PU* treatment significantly attenuates these effects.

### 3.2 *PU* Suppresses Renal Inflammation in Cisplatin‐Induced AKI Mice

**FIGURE 2:**
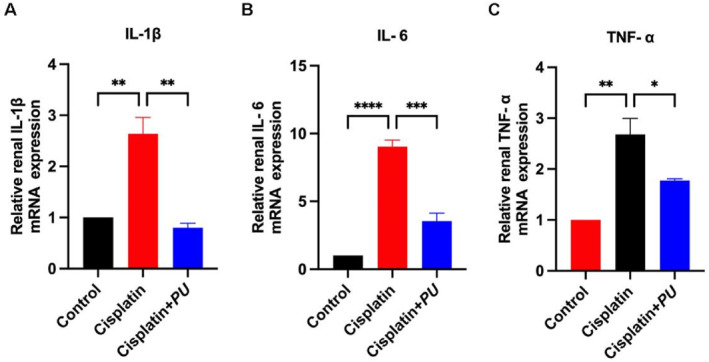
Examination of inflammatory factors level in the control group, cisplatin group, and cisplatin+*PU* group. (A‐C) qPCR detects the expression of inflammatory factors (IL‐1β, IL‐6, and TNF‐α). The administration of *PU* resulted in the suppression of elevated expression levels of inflammatory factors observed in the cisplatin+*PU* group. *p < 0.05, **p < 0.005, ***p < 0.0005, and ****p < 0.0001. qPCR, Quantitative polymerase chain reaction; IL‐1β, interleukin‐1β; IL‐6, interleukin‐6; TNF‐α, Tumor Necrosis Factor Alpha.

Renal interstitial inflammation is well recognized as a key contributor to cisplatin‐induced AKI. By using qPCR to measure the levels of interleukin‐1β (IL‐1β), interleukin‐6 (IL‐6), and tumor necrosis factor‐α (TNF‐α), we found that, in comparison to the cisplatin group, *PU* intervention greatly mitigated the production of inflammatory markers (Figure 2 A‐C).

### 3.3 *PU* Lessens Cisplatin‐Induced Apoptotic Cell Death

**FIGURE 3:**
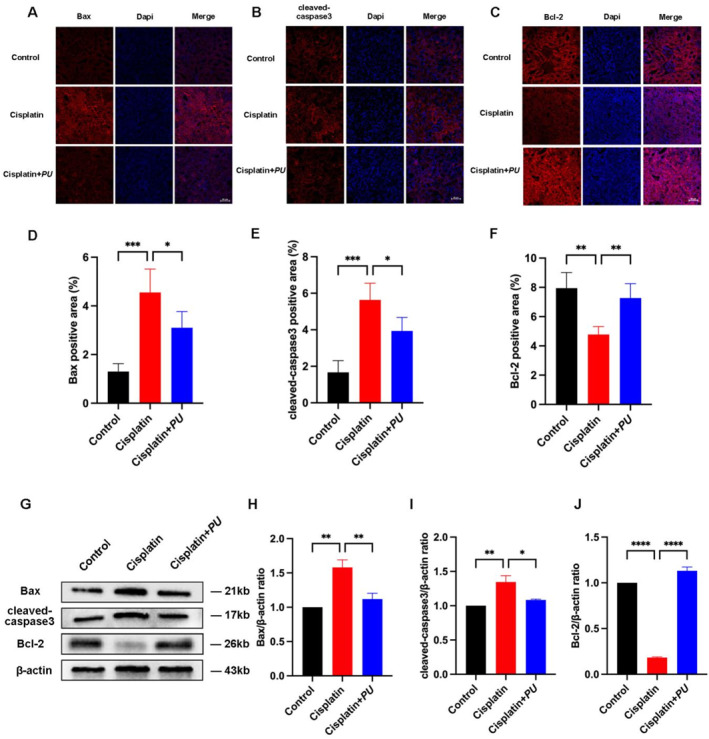
Examination of apoptosis‐related markers in the control group, cisplatin group and cisplatin+*PU* group. (A‐C) Immunofluorescence of apoptosis‐related markers (Bax, cleaved‐caspase3, and Bcl‐2) in the kidney sections in each group (scale bar: 50 μm). (D‐F) Numerical evaluation of immunofluorescence staining for apoptosis‐related markers (Bax, cleaved‐caspase3, and Bcl‐2) in each group. (G) Western blot of apoptosis‐related markers (Bax, cleaved‐caspase3, and Bcl‐2) in each group. (H‐J) Relative expression of apoptosis‐related markers (Bax, cleaved‐caspase3, and Bcl‐2) detected by Western blot. *p < 0.05, **p < 0.005, ***p < 0.0005, and ****p < 0.0001. Bax, Bcl2‐Associated X protein; Bcl‐2, B cell leukemia/lymphoma two.

Apoptosis is acknowledged as another significant molecular process implicated in the onset and development of AKI, in addition to inflammation and oxidative stress. *PU* inhibited cisplatin‐AKI‐induced apoptosis in kidney cells by measuring levels of the apoptosis‐associated proteins B cell leukemia/lymphoma two (Bcl‐2), Bcl2‐Associated X protein (Bax), and cleaved‐caspase3 using Western blot and immunofluorescence (Figure 3). In contrast to the control group, our findings indicated that the cisplatin group had higher levels of Bax and cleaved‐caspase3 expression and lower levels of Bcl‐2 expression. However, *PU* pretreatment completely corrected these alterations. These findings imply that *PU* can reduce the amount of cisplatin‐induced apoptosis in mice.

### 3.4 *PU* Decreases Cisplatin‐Induced Renal Oxidative Stress

**FIGURE 4:**
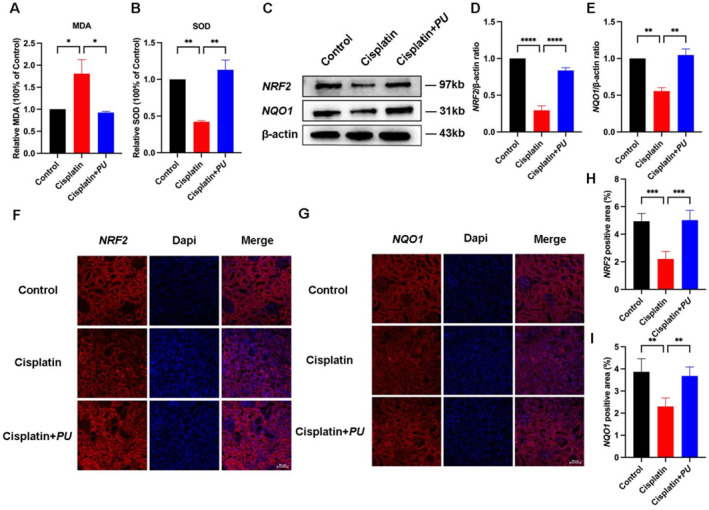
Examination of oxidative stress indictors and related pathway markers in the control group, cisplatin group, and cisplatin+*PU* group. (A and B) Levels of MDA and SOD in each group. The administration of *PU* resulted in a decrease in the expression of MDA and an increase in the expression of SOD. (C) Relative expression of *NRF2*/*NQO1* detected by western blot. (D and E) Western blot of *NRF2*/*NQO1* in each group. (F and G) Immunofluorescence of *NRF2*/*NQO1* in the kidney sections in each group (scale bar: 50 μm). (D‐F) Numerical evaluation of immunofluorescence staining for *NRF2* and *NQO1*. *p < 0.05, **p < 0.005, ***p < 0.0005, and ****p < 0.0001. MDA, malondialdehyde; SOD, superoxide dismutase.

According to an analysis of *PU*'s effects on oxidative stress, we know that the antioxidant system of kidneys was strengthened and oxidative stress caused by cisplatin was greatly reduced. Figure 4A, B show that cisplatin significantly decreased antioxidant enzyme SOD levels while significantly increasing lipid peroxide MDA levels after treatment. As a result of treatment with *PU*, MDA expression was decreased and renal SOD activity was restored. We investigated whether *PU* had an impact on the expression of *NRF2*/*NQO1* as *NRF2* has the ability to control the expression of antioxidant enzymes which has been shown in many studies [17, 18]. Cisplatin dramatically reduced the expression of *NRF2* and its downstream target gene *NQO1*, according to the results of the Western blot study (Figure 4C–E). The administration of *PU*, however, overturned this pattern and increased their expression. Immunofluorescence analysis further supported the previous findings that *PU* administration can restore the decreased expression of *NRF2* and *NQO1* induced by cisplatin (Figure 4F, G).

## 4 Discussion

AKI represents a critical global health concern, accounting for 1.7 million deaths annually and posing substantial medical and socioeconomic challenges [19]. Drug‐induced AKI accounts for 19%–26% of all hospital admissions, attributed to nephrotoxic agents such as aminoglycosides, vancomycin, nonsteroidal antiinflammatory drugs, cyclosporine, tacrolimus, and, notably, cisplatin [20, 21]. Given cisplatin's prevalent nephrotoxic side effects in cancer therapy, identifying effective preventive strategies against cisplatin‐induced nephrotoxicity is imperative. Notably, *Phyllanthus niruri* has shown to significantly improve renal function in diabetic rats through mitigating oxidative stress, inflammation, fibrosis, and apoptosis [22]. The pharmacological attributes of *Phyllanthus* species, particularly *PU*, have garnered increasing scientific interest, prompting this investigation into its renal protective potential against cisplatin‐induced AKI.

The established cisplatin‐induced AKI mouse model facilitated the examination of *PU*'s protective efficacy. Cisplatin administration at 20 mg/kg resulted in marked renal damage as evidenced by elevated Scr and BUN level, corroborated by histopathological observations [23, 24]. However, pre‐treatment with *PU* (250 mg/kg) significantly ameliorated these indicators of renal injury, suggesting a protective action of *PU* against cisplatin‐induced AKI.

Further analysis explored *PU*'s impact on oxidative stress and apoptotic signaling pathways, pivotal in AKI pathophysiology and possibly chronic kidney disease progression [25, 26, 27]. Cisplatin‐induced nephrotoxicity's inflammatory response, characterized by elevated proinflammatory cytokines and chemokines production [28], underscores cisplatin‐induced AKI's inflammatory nature. This investigation revealed that cisplatin treatment upregulated TNF‐α, IL‐1β, and IL‐6 mRNA expression in renal tissue, and a trend effectively counteracted by *PU* administration, thus delineating a potential anti‐inflammatory mechanism of *PU* in alleviating cisplatin‐induced AKI.

Apoptotic signaling, crucial in cisplatin‐induced renal tubular cell death, with cisplatin enhancing Bax expression while Bcl‐2 inhibits Bax expression to attenuate apoptosis, emerges as another area impacted by *PU* [29, 30, 31, 32]. Our data demonstrate *PU*'s ability to markedly diminish cisplatin‐induced apoptosis by modulating Bax, Bcl‐2, and cleaved‐caspase3 expression, hinting at *PU*'s role in apoptosis regulation.

Oxidative stress, driven by an imbalance between pro‐oxidants and antioxidants, constitutes a significant aspect of cisplatin‐induced AKI pathology [33]. This study observed *PU*'s capacity to elevate SOD levels while reducing MDA levels, signifying oxidative stress alleviation. The *Nrf2* and *NQO1* pathway's pivotal role in managing oxidative stress and inflammation further illuminates *PU*'s protective mechanism, facilitating kidney defense against cisplatin‐induced damage [34, 35, 36, 37, 38]. Notably, activating *Nrf2* not only curtails oxidative stress but also intervenes in the Bax/Caspase‐3 apoptotic pathway, consolidating *PU*'s antioxidative prowess [39, 40, 41, 42].

In fact, *PU* is widely used in clinics. For example, *PU* and its preparations are effective in the treatment of chronic hepatitis B, and the long‐term use of *PU* is very effective. In addition, a decoction with *PU* as its main component can delay and prevent the progression of fibrosis in patients with hepatitis B. Because of the limited space of the article, the toxicology is not discussed too much. The interplay between *PU*'s *NRF2*/*NQO1* pathway activation and Bax/c‐caspase3 signaling pathway modulation provides a plausible explanation for its efficacy against cisplatin‐induced AKI, warranting further exploration to elucidate *PU*'s underlying mechanisms in AKI management. However, there are limitations to our study. We use *PU* water extract and further research is needed to determine which components play a major role. Moreover, although we observed a positive effect of *PU* on AKI, other pathways and mechanisms that may be involved simultaneously have not been complete excluded.

In conclusion, this is the first systematic assessment of the protective effect of *PU* in cisplatin‐induced AKI models. A variety of biochemical and molecular biological methods were used to preliminarily reveal the multiple protective mechanisms of *PU,* which provide a new research direction and scientific basis for the application of Chinese herbal medicine in kidney protection. The finding enriches our understanding of AKI treatment strategies and the application of traditional Chinese medicine in addressing drug‐induced nephrotoxicity, and provides a new perspective and theoretical basis for the clinical management of AKI.

## Author Contributions

D.Z. contributed to the conception and design, Q.G. and P.L. contributed to the investigation and writing of the original draft, M.C., and Y.Y. contributed to the collection and processing of the data, C.L. and L.S. provided technological guidance and revised the manuscript critically for important intellectual content, and X.L. and G.W. conducted the statistical analysis. All authors have read and agreed to the published version of the manuscript.

## Ethics Statement

All experimental procedures involving animals were conducted in accordance with the Basel Declaration and were approved by the ethics committee of Chongqing Medical University (approval No. CHCMU‐IACUC2022042900, approval date 31 March 2022).

## Consent

All authors approved the final manuscript and the submission to this journal.

## Conflicts of Interest

The authors declare no conflicts of interest.

## Supporting information

Supporting Information S1

## Data Availability

The data that support the findings of this study are available from the corresponding author upon reasonable request.
