# Supplementary material for: Therapeutic Effects of Phyllanthus urinaria L on Cisplatin‐Induced Acute Kidney Injury: Anti‐Inflammatory, Anti‐Apoptotic, and Anti‐Oxidative Action
Source: Pediatr Discov. 2025 May 15;3(4):e70000. doi: 10.1002/pdi3.70000 (PMC12753014; doi:10.1002/pdi3.70000)
Supplement: Supplementary file 1 — Supporting Information S1 [file PDI3-3-e70000-s001.docx]

**Supplementary** **Table 1.** primer sequences for qPCR.

| **Gene** | **Forward Primer Sequence (5**′**–3**′**)** | **Reverse Primer Sequence (5**′**–3**′**)** |
| --- | --- | --- |
| IL-6(Ms)  IL-1β(Ms)  TNF-α(Ms)  GAPDH(Ms) | AACCGCTATGAAGTTCCTCTCTG CACTACAGGCTCCGAGATGAACAAC  GCCTCCCTCTCATCAGTCTA AGGTCGGTGTGAACGGATTTG | TGGTATCCTCTGTGAAGTCTCCT TGTCGTTGCTTGGTTCTCCTTGTAC  GGCAGCCTTGTCCCTG  TGTAGACCATGTAGTTGAGGTCA |
